# Use, abuse and misuse of notes to file

**DOI:** 10.4103/2229-3485.76289

**Published:** 2011

**Authors:** Aditi Hazra

**Affiliations:** *Clinical Research Consultant, 1103, Glen Croft, Hiranandani Gardens, Powai, Mumbai, Maharashtra, India*

**Keywords:** Documentation, essential documents, file notes/notes to file, source documents

## Abstract

A quick and easy solution to the absence of adequate documentation during clinical trial conduct is the use of notes to file. Over the years, the use of notes to file has evolved from a last resort solution to a common working practice amongst clinical teams, bordering on misuse and abuse of this tool. This article explores this evolution from the perspective of an independent observer.

## INTRODUCTION

A decade and a half of conducting global clinical trials in India has taught us many things, especially the importance of documentation in research. The nuances of the extent and the ways to document clinical trial data, however, continue to elude us. One of the most common questions asked by investigators participating in clinical trials is: How much documentation is enough? Their frustration has to do with the fact that no amount of detailed documentation seems to satisfy monitors, auditors and inspectors. There are several instances where, in the absence of an adequate documented explanation, the investigators are asked to generate a note to file (NTF), which contains a detailed explanation. An example would be a case where the site staff has not followed the protocol-specified investigational product dosing schedule for several subjects. In such cases, it is common to see an NTF in the site master file explaining this deviation and the reason for the same.

Initially, this practice of using NTFs to document deviant processes began when the site team and the monitoring team could not find an appropriate place to document certain issues. However, nowadays, little or no effort goes into even considering whether issues can be documented elsewhere and NTFs are generated, without batting an eyelid, to document everything ranging from banal logistic issues to those of serious non-compliance.

## THE USE AND MISUSE

The indication that the excessive use of NTFs is not a desirable practice should come from the fact that no guidelines[1,2] mention their usage. One possibility is that their usage is more a solution to comply with guidelines rather than instructions given in the guidelines. An acceptable use of an NTF in clinical trial conduct documentation would be as seen in the following example. A mid-sized pharmaceutical conducting a late Phase 3 clinical trial decides not to release an annual revision of the Investigators’ Brochure (IB) as there are very little new safety data to merit a new IB version. Their solution is to document this decision in the form of an NTF signed by the study clinician and share it with all members of the study team and all investigators working on trials with that molecule.

In general, NTFs are used in the following instances:

- To document the reason for missing, delayed or erroneous documents in the clinical trial master file or in the site master file.
- To explain protocol deviations or investigator site practices that are different from the norm or from what is prescribed in the protocol.

During the review of trial master files, one often comes across an additional way that NTFs are used to document that certain sections of the files are not applicable. For example, for an ongoing study, the file will contain a section for the clinical study report, which is expected to be blank; however, it is a common but unnecessary practice to place a file note stating that this section is not applicable. It is, however, acceptable and recommended that if contents of a particular section of the file are placed in a separate file, this should be indicated via a file note in that section.

One of the most common ways to misuse an NTF is to use it in place of source documents. This happens when the investigator site staff misses out essential information about trial conduct for subjects in their respective source documents or medical records. It can even happen when the documentation in the source document needs further clarification. In such cases, all additional information and clarifications are documented on NTFs instead of entering them in the source documents directly or explaining them in the monitoring visit reports. For example, if the site staff omit to document the pill counts done after every visit for one or more subjects, this is tabulated and entered for all those subjects together in an NTF placed in the site master file retrospectively.

The misuse of NTFs has been cited in warning letters issued by the US-FDA. In these cases, the inspectors from the US-FDA Department of Scientific Investigations (DSI) have admonished sponsors for documenting important events in a clinical trial simply by generating a “Memo-to-File”. One such example is illustrated in the warning letter issued to Sanofi-Aventis in October 2007,[3] where serious issues of non-compliance in the informed consent process were resolved by documenting the violations in NTFs. The inspector stated in this letter “*memos to file are inadequate to address the falsification (backdating) of study documents”*. A more recent example can be seen in the warning letter issue to Johnson and Johnson PRD in 2009,[4] where an NTF was used to override the decision made to select an investigator site with a previous history of non-compliance.

The need to document long drawn-out explanations in the NTFs stems from our need to assign a reason for why and how a deviation happened and to document it. It is perfectly acceptable that during the course of clinical trial conduct, there are accidental deviations from the protocol. These do not always need to be explained and analyzed via NTFs as long as the site team and the monitoring team realize the error and ensure that it does not get repeated. The more important action item, rather than generating NTFs, should be to ensure training of all parties involved and assessing and communicating the impact of the deviation to the clinical team.

An interesting viewpoint for the use of NTFs to document site errors or deviations in the protocol made by the investigator site team surfaced during an investigator site audit. The monitor would insist that the Principal Investigator (PI) must sign all NTFs as a punitive action so that such errors do not occur again and to document that he was taking responsibility for these errors.

## THE ABUSE

The realization that the use of NTFs has evolved to abuse hits when we come across working practices and guidance documents supporting the use of NTFs. In many cases, even version dated templates are provided for creating NTFs and a separate section in the site or trial master file is created to file them. There are organizations where NTFs are submitted as attachments to monitoring reports by the site monitors to prove that they have successfully closed out site issues during their monitoring visits. This not only sends the message to the monitors and site staff that NTFs are acceptable but also makes it a preferable and a convenient means to document issues.

Now you may wonder why this is of concern and what is the impact of generating numerous NTFs when their only purpose is to clearly document events in a clinical trial. The primary concern is that it is often used as a solution to issues arising at sites. If an investigator site is cited for having too many protocol deviations, it is not uncommon for them to retort that NTFs were generated to explain the deviations. They do not realize that the documented explanation does not take away from the fact that deviations have occurred.

Also, when the use of NTFs to document issues at the site is a common practice, there arise situations where the same issue is explained in the source document, in the monitoring visit report, in a letter to the ethics committee and also in a separate NTF. This not only overstates the obvious, but also makes the site staff sound excessively defensive and wastes precious time of all those who are involved in creating, printing and signing the NTF. Alternatively, it can also happen that since these issues are already documented in the NTF, they often get omitted from places where they should be documented like the monitoring visit reports and site correspondence.

## THE ALTERNATIVES

It is only fair that some alternative solutions are provided while strongly recommending avoiding the use of NTFs. This is not difficult as the information documented on NTFs logically belongs to one or more of following locations:

- The subject’s source document file.
- The monitoring or site visit report where that issue was first noted and discussed with the site staff.
- The correspondence between the site staff and the monitoring team, internal correspondence within the sponsor/ contract research organization (CRO) teams or correspondence with the ethics committee and regulators.

In very rare instances, it would be necessary to generate an NTF if the information does not belong to any of the above categories. But it is highly likely that these instances would be exceptions rather than the norm.

## CONCLUSION

The institutionalization of the use (or misuse) of NTFs does not reflect well on our ability to understand and interpret the spirit of clinical research guidelines. It indicates that we are increasingly becoming incapable of exercising judgment and playing it safe by documenting almost everything that happens during the conduct of a trial in NTFs rather than where they should logically be documented. It is important that all of us stop and think before generating the next NTF, whether it is necessary to do so or can the documentation of this issue be done more appropriate elsewhere.
